# Ontario Veterinary Association

**Published:** 1897-02

**Authors:** C. H. Sweetapple

**Affiliations:** Secretary


### ONTARIO VETERINARY ASSOCIATION.

THE annual meeting was held in the Veterinary College, Toronto, on December 22, 1896. The meeting was opened with the President, Mr. William H. Hopkins, of Green River, Ont., in the chair. In his opening address the President made a few well-chosen remarks on the benefits to be derived, both individually and to the profession at large, by attendance at these annual gatherings, and he hoped to see still greater interest taken in them in the future by all members of the profession in this province.

The reports of the secretary, treasurer, and auditor were read and adopted.

A motion was carried that the initiation fee be reduced to $3.00 and the annual dues to $1.00.

The following new members were duly proposed and accepted: Mr. James Mayhew, V.S., of Cookstown, Ont.; Mr. S. Lawson, V.S., of Acton, Ont.; Mr. Lawson, V.S., of Dundas, Ont.; Mr. R. F. Golden, V.S., of Windsor, Ont.; Mr. James Gregg, V.S., of Little Britain, Ont.; Mr. J. H. Reed, V.S., of Guleph, Ont., and Mr. A. R. Metcalf, V.S., of Vankleek Hill, Ont.

A discussion then ensued on the proportion of the fines imposed, under the provisions of the recent Veterinary Act, that should be paid over to the prosecutors, these fines being the property of this Association, and it was resolved that as the Association did not wish to be pecuniarily benefited by the fines, but wished to protect the profession, "that the greater part should be paid over to the prosecutor, a small proportion only to be retained to defray necessary expenses.

The election of officers for the ensuing year then took place with the following result: Major Lloyd, President; Mr. S. Sisson, 1st Vice-President; Mr. H. S. Wende, 2d Vice-President; Mr. Sweetapple, Secretary and Treasurer; Directors: Mr. W. Shaw, Mr. W. Gibb, Mr. J. W. Faskine, Mr. W. J. Wilson, Mr. John Wende, Mr. W. Steele, Mr. W. Cowan, and Mr. R. F. Golden; Auditors, Mr. C. Elliott and Mr. J. D. O'Neil. Delegates to the Western Fair Association, Messrs. J. H. Wilson, Secretary, and J. D. O'Neil. Delegates to the Industrial Fair Association, Prof. A. Smith and Major Lloyd.

The retiring President having vacated the chair in favor of the President-elect, a hearty vote of thanks was tendered to Mr. H. Hopkins for his able conduct in the chair and the interest he had taken in the Association during his term of office.

Major Lloyd, on assuming the chair, thanked the members cordially for the honor conferred on him, and promised to do all in his power for the best interests of the Association. Among other matters he urged upon all the members the benefits that might accrue if each one were to give an account at our next meeting of some case of special interest that he had met with in his practice during the past year, even if it were only one case; but he hoped everyone would endeavor to contribute something.

Prof. J. H. Reed, of the Guelph Agricultural College, gave an interesting account of some cases of cerebro-spinal meningitis that he had met with; he mentioned particularly the paralysis of the muscles of deglutition as one of the symptoms. He had had bacteriological examinations made of the water used, and he believed the cause of the disease to have been pathogenic bacteria in the water. Hyposulphite of soda and also nux vomica had been used in the medicinal treatment of the disease.

Mr. R. F. Golden, V.S., of Windsor, Ont., gave an account of the disease among swine that had existed in the county of Essex. He described the symptoms he had observed, also the post-mortem appearances. He mentioned that in some cases the bowels showed more indications of disease than the lungs, while in other cases the reverse conditions were apparent, and frequently both the lungs and bowels were implicated.

Mr. Gibb, V.S., of Stratford, Ont., read an excellent paper on the "Value of Action and Position as Indications of Lameness in the Horse, and the Diagnosis of its Location." Interesting discussions took place at the close of each, in which many members participated. A hearty vote of thanks was tendered to each of the gentlemen mentioned for their valuable contributions. A discussion then came up, in which many took part, on the need for better legal protection to the profession and the best mode to be adopted for endeavoring to secure it, and it was strongly urged that all members of the profession should act in unison, as only by that means can we hope to attain the object in view.

Several other matters of interest were brought forward and discussed.

The sum of twenty-five dollars was appropriated for a medal to be competed for by the students of the Ontario Veterinary College at the approaching spring examinations, and the meeting adjourned.

C. H. Sweetapple,
Secretary.

